# Authoritative Textbook-Augmented Large Language Models for High-Altitude Public Health Medical Education in the Xizang Autonomous Region: Cross-Sectional Comparative Evaluation Study

**DOI:** 10.2196/92852

**Published:** 2026-06-16

**Authors:** Kun He, Qiming Xiao, Wangyang Chen, Lisha Jing, Yabing Wang, Shuai Li, Daiyu Yang, Hemiao Xu, Ke Pang, Ruoyu Xiao, Zhashilamu Liu, Deji Zhuoga, Ruxuan Chen, Jingyi Li, Long Chang, Yangzhong Zhou, Zhe Zhang, Ran Li, Lujing Ying, Rutong Li, Hongwei Wang, Xin Yin, Ge Zhen, Siyi Cai, Qijun Shan, Qiang Wang, Danzeng Zhuoga, Ciren Yangjin, Gesang Luobu, Tu Ji, Dong Wu

**Affiliations:** 1 Department of Gastroenterology, State Key Laboratory of Complex Severe and Rare Diseases, Peking Union Medical College Hospital, Shuaifuyuan, No.1, Dongcheng District, Beijing, Beijing, 100730, China, 86 010-69151591; 2 Department of Gastroenterology, Tibet Autonomous Region People's Hospital, Lhasa, Tibet, China; 3 Chinese Academy of Medical Sciences & Peking Union Medical College, Beijing, Beijing, China; 4 Department of Endocrinology, Beijing Friendship Hospital, Beijing, Beijing, China; 5 Medical College, Xizang University, Lhasa, Tibet, China; 6 Department of Pulmonary and Critical Care Medicine, Peking Union Medical College Hospital, Beijing, Beijing, China; 7 Department of Cardiology, State Key Laboratory of Complex Severe and Rare Diseases, Peking Union Medical College Hospital, Beijing, Beijing, China; 8 Department of Hematology, Peking Union Medical College Hospital, Beijing, Beijing, China; 9 Department of Rheumatology and Clinical Immunology, National Clinical Research Center for Dermatologic and Immunologic Diseases, Peking Union Medical College Hospital, Beijing, Beijing, China; 10 Department of Neurology, Peking Union Medical College Hospital, Beijing, Beijing, China; 11 Department of Endocrinology, Key laboratory of Endocrinology of National Health Commission, Peking Union Medical College Hospital, Beijing, Beijing, China; 12 Department of Gastroenterology, Air Force Medical Center, Chinese People's Liberation Army, Beijing, Beijing, China; 13 Department of Orthopedics, Peking Union Medical College Hospital, Beijing, Beijing, China; 14 Information Center, Peking Union Medical College Hospital, Beijing, Beijing, China; 15 High Altitude Medical Research Institute, Tibet Autonomous Region People's Hospital, Lhasa, Tibet, China; 16 Institute of Basic Medical Sciences, Chinese Academy of Medical Sciences & Peking Union Medical College, Beijing, Beijing, China

**Keywords:** public health medical education, large language model, authoritative textbooks, retrieval-augmented generation, high altitude

## Abstract

**Background:**

Public health medical education is increasingly important in the low-resource, high-altitude Xizang Autonomous Region (Tibet). Traditional authoritative textbooks do not meet modern needs for accessibility and interactivity, whereas general large language models (LLMs) may hallucinate in specialized medical domains. Developing specialized LLMs for low-resource regions is also expensive and difficult.

**Objective:**

This study aimed to explore a novel approach to high-altitude public health medical education in the low-resource Xizang Autonomous Region that integrates modern LLMs and authoritative textbooks, using a comprehensive benchmark evaluation across multiple dimensions and retrieval-augmented generation (RAG) technology.

**Methods:**

We conducted a 2-stage cross-sectional comparative evaluation study to benchmark publicly available LLMs and evaluate the added value of textbook-augmented retrieval under standardized generation settings and blinded expert assessment. First, 4 publicly available LLMs (GPT-5.2 [OpenAI], Gemini 3.0 Pro [Google], DeepSeek R1 [DeepSeek], and Tencent HY 2.0 [Tencent]) were benchmarked using an 80-question benchmark on high-altitude public health medicine developed by authoritative medical specialists. Each question was asked 3 times, yielding 960 outputs; first responses (n=320) were scored under blinded conditions by 2 independent 8-member physician panels. A clinically weighted evaluation of multidimensional first-response scores (including comprehensiveness, accuracy, clarity, and relevance) and a composite consistency metric (including semantic similarity and algorithmic similarity) was administered. Second, 4 specific and prevalent authoritative textbooks on high-altitude public health medicine*—Ward, Milledge and West’s High Altitude Medicine and Physiology, High Altitude Medicine: A Case-Based Approach, High Altitude Medicine,* and *High Altitude Medical Protection*—were deployed as the external knowledge base for the evaluation-optimized model. Statistical analyses included Spearman ρ, Cronbach α, intraclass correlation coefficients, Friedman tests with Dunn multiple comparisons, and paired Wilcoxon signed-rank tests. The significance threshold was set at α=.05.

**Results:**

DeepSeek R1 was selected as the optimal base model for achieving the highest weighted score (5.61/10.00), followed by GPT-5.2 (5.51/10.00), Gemini 3.0 Pro (5.39/10.00), and Tencent HY 2.0 (4.71/10.00). The deployed retrieval-augmented model integrating the authoritative textbooks and the optimal LLM DeepSeek R1, HPHME-Xplus-RAG, achieved remarkable improvement in multidimensional scores compared to baseline DeepSeek R1 (median 8.00 [IQR 7.88‐8.00] vs median 7.63 [IQR 7.38‐7.88]; *P*<.001, r_rb=0.68, indicating a large effect).

**Conclusions:**

Integrating authoritative textbooks with an evaluation-optimized general LLM through an RAG framework showed strong performance for medical education in the low-resource Xizang Autonomous Region. Unlike prior studies that mainly evaluated general LLMs or used clinical guidelines to build RAG systems for diagnosis and treatment, this study used authoritative textbooks for the broader, guideline-scarce field of public health medical education. This work provides a replicable workflow—domain-authoritative knowledge+RAG+model optimization and evaluation—for low-resource settings, with practical implications for medical instructors and students, hospitals, and public health services seeking cost-effective, convenient, and trustworthy educational support.

## Introduction

High-altitude public health medical education is essential for protecting individual well-being and regional public health in plateau settings. High-altitude regions, including the Xizang Autonomous Region (Tibet), are culturally and ecologically significant and have attracted increasing attention because plateau exposure poses substantial physical and psychological challenges to both residents and visitors [1-3]. Existing evidence shows that high-altitude environments may affect multiple physiological and behavioral domains, including cardiovascular function, cognitive performance, sleep quality, blood pressure regulation, neurological symptoms, musculoskeletal adaptation, inflammatory and immune responses, and mental health [4-10]. These multisystem effects can impair daily functioning, increase health risks during travel, work, and long-term residence, and create practical demands for early recognition, prevention, and self-management. Therefore, timely public health medical education is needed to raise awareness and promote preventive behaviors among medical instructors and students, frontline health care workers, residents, and travelers.

However, public medical education in this setting faces substantial structural constraints, particularly in low-resource regions such as the Xizang Autonomous Region. Studies have shown insufficient general practitioner training and limited continuing medical education opportunities [11-13]. Local reports have also described difficulty accessing standardized lectures and textbooks because of limited educational resources, as well as shortages of qualified teachers and the limited relevance of standardized teaching materials to plateau-specific conditions [14,15]. In addition, existing formal guidelines and consensus statements in high-altitude medicine are fragmented and largely focused on acute altitude illness, travel medicine, or selected subgroups, while comprehensive guidance specifically addressing high-altitude public health medicine remains scarce [16-20].

Although authoritative textbooks have long served as the primary medium for medical education in this field and encapsulate years of accumulated expertise, conventional materials alone increasingly struggle to meet modern expectations for accessibility, efficiency, and interactivity. Large language models (LLMs) have rapidly advanced driven by advances in deep learning and the Transformer architecture, supporting a broad range of medical tasks, including medical education, clinical decision-making, specialty-specific question answering, and diagnostic reasoning, although their performance varies substantially across specialties, tasks, and evaluation settings [21-39]. Despite this growing body of literature, evidence for LLM use in high-altitude medical education remains scarce. Therefore, although LLMs show considerable promise in spreading public health information, especially in settings where traditional outreach is limited, their role in high-altitude public health medical education remains insufficiently studied, with domain-specific accuracy and hallucinations probably being a key limitation [40,41]. Also, developing specialized large models for low-resource domains and regions is prohibitively expensive and difficult. This contrast underscores the need for hybrid approaches that combine the strengths of both paradigms.

Retrieval-augmented generation (RAG) offers such a bridge. By integrating external, authoritative sources into the generative process, RAG allows an LLM to retrieve verified information before producing responses, providing a cost-effective approach compared to developing a new proprietary LLM for low-resource regions [42-47]. In high-altitude public health medical education, RAG has the potential to merge authoritative content with the flexibility of LLMs to deliver reliable and personalized information. This study aims to evaluate a novel approach that integrates the accuracy of authoritative textbooks with the efficiency and reasoning capacity of an evaluation-optimized LLM using the RAG mechanism for high-altitude public health medical education in this low-resource setting.

## Methods

### Study Design

**Figure 1.**
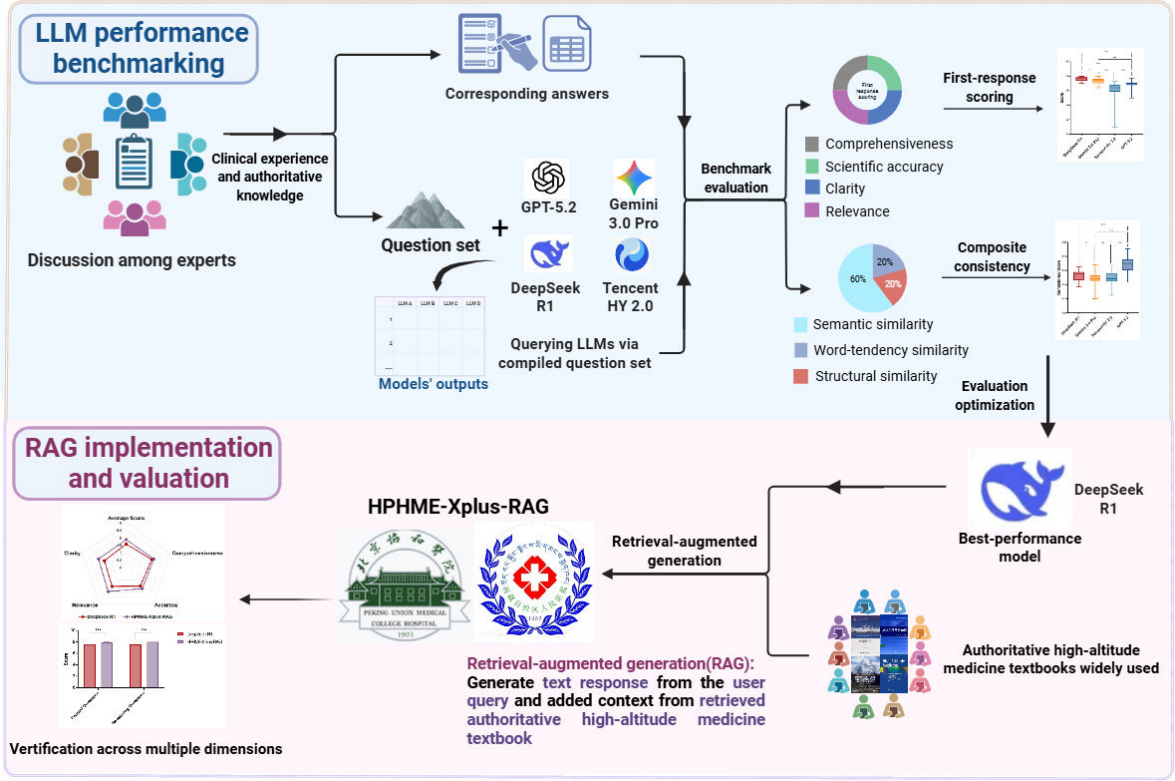
Study design of a 2-stage cross-sectional comparative evaluation study on large language models for high-altitude public health medical education in the Xizang Autonomous Region (Tibet), China. LLM: large language model; RAG: retrieval-augmented generation; HPHME-Xplus-RAG: High-Altitude Public Health Medical Education–Xplus (Xiehe in Chinese for Peking Union Medical College Hospital and Xizang for People’s Hospital of Xizang Autonomous Region)–Retrieval-Augmented Generation.

This was a 2-stage cross-sectional comparative evaluation study conducted to select an optimal LLM for the RAG architecture and to evaluate its performance in high-altitude public health medical education. First, the performance of 4 extensively adopted publicly available LLMs in answering questions on high-altitude health medical education was evaluated. Based on the weighted results of the multidimensional first-response scores and composite consistency metrics with the weighting ratio determined by the clinical needs assessment, the LLM with the best performance was selected. Second, a retrieval corpus constructed from the specific and prevalent authoritative textbooks was deployed as an external data source for the selected LLM to develop a RAG architecture for medical education on high-altitude health, and the performance improvement of the RAG architecture compared to the LLMs was verified (Figure 1).

### Data Collection

A total of 80 questions and corresponding reference answers were developed through an expert-driven, iterative process. Multidisciplinary specialists from Peking Union Medical College Hospital (PUMCH) and People’s Hospital of Xizang Autonomous Region collaboratively proposed and refined candidate questions on the basis of real-world diagnostic, therapeutic, preventive, and health education scenarios encountered in high-altitude practice. Rather than using random question sampling, we adopted a purposive selection strategy to ensure that the benchmark represented the major and practice-relevant domains of high-altitude public health medicine.

**Figure 2.**
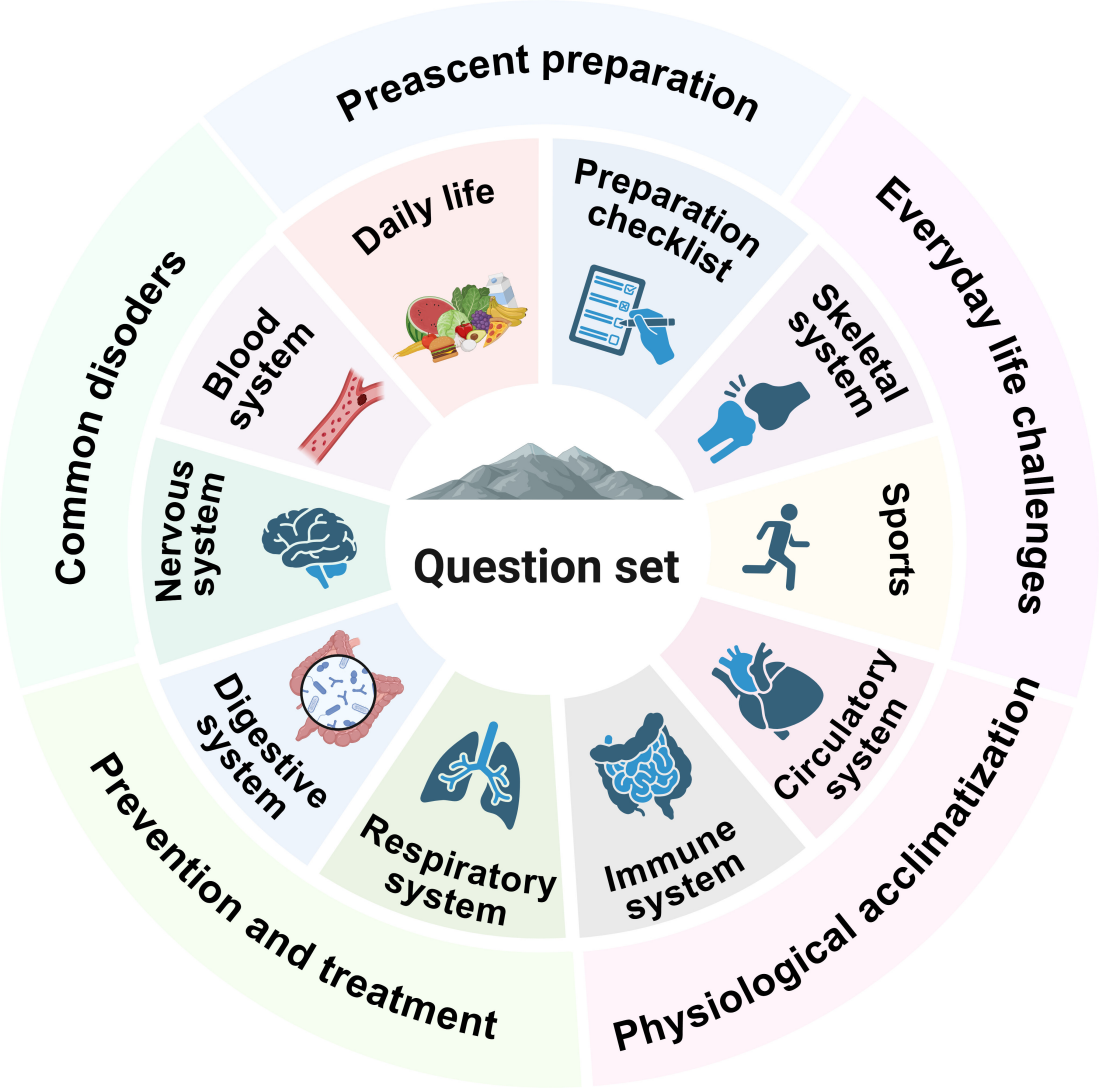
Composition of the 80-question benchmark used in this cross-sectional comparative evaluation study of large language models for high-altitude public health medical education.

During question set construction, particular attention was paid to broad domain coverage across the multisystem health effects of hypobaric hypoxia and plateau exposure. The final benchmark therefore included respiratory, cardiovascular, gastrointestinal, neurological, hematological, musculoskeletal, and infectious disease topics, as well as lifestyle, nutrition, and psychological acclimatization issues. In this way, the benchmark was designed to reflect the interdisciplinary nature of high-altitude public health medicine, spanning clinical medicine, physiology, prevention, and behavioral health (Figure 2). The preliminary question set was subsequently reviewed and validated by Xizang-aid physicians from PUMCH with direct experience in high-altitude health adaptation in order to confirm its contextual relevance, content breadth, and practical representativeness for the target setting. Each question was accompanied by an expert-defined reference answer, which served as the “gold standard” with a full score of 10 out of 10. These standard answers were based on authoritative sources, including clinical guidelines, textbooks, and medical specialists’ consensus.

Four LLMs—GPT-5.2 (OpenAI), Gemini 3.0 Pro (Google), DeepSeek R1 (DeepSeek), and Tencent HY 2.0 (Tencent)—were selected a priori because they were publicly available, stably accessible during the study period, and representative of major contemporary LLM families across both international and Chinese developer ecosystems [48-51]. Restricting stage 1 to standalone general-purpose models allowed a controlled comparison of intrinsic model performance before subsequent RAG augmentation. All models were accessed through their respective application programming interfaces under standardized conditions, with temperature set to 0.1 and top_p to 0.9 during generation to ensure result comparability, leaving all other hyperparameters at their developer defaults.

Each question was posed 3 times to each LLM, generating 3 independent responses per model. Only the first response was scored to simulate real-world, on-demand information retrieval without iterative prompting, while all 3 were used for consistency analysis.

### Quantitative Evaluation of First Responses

**Figure 3.**
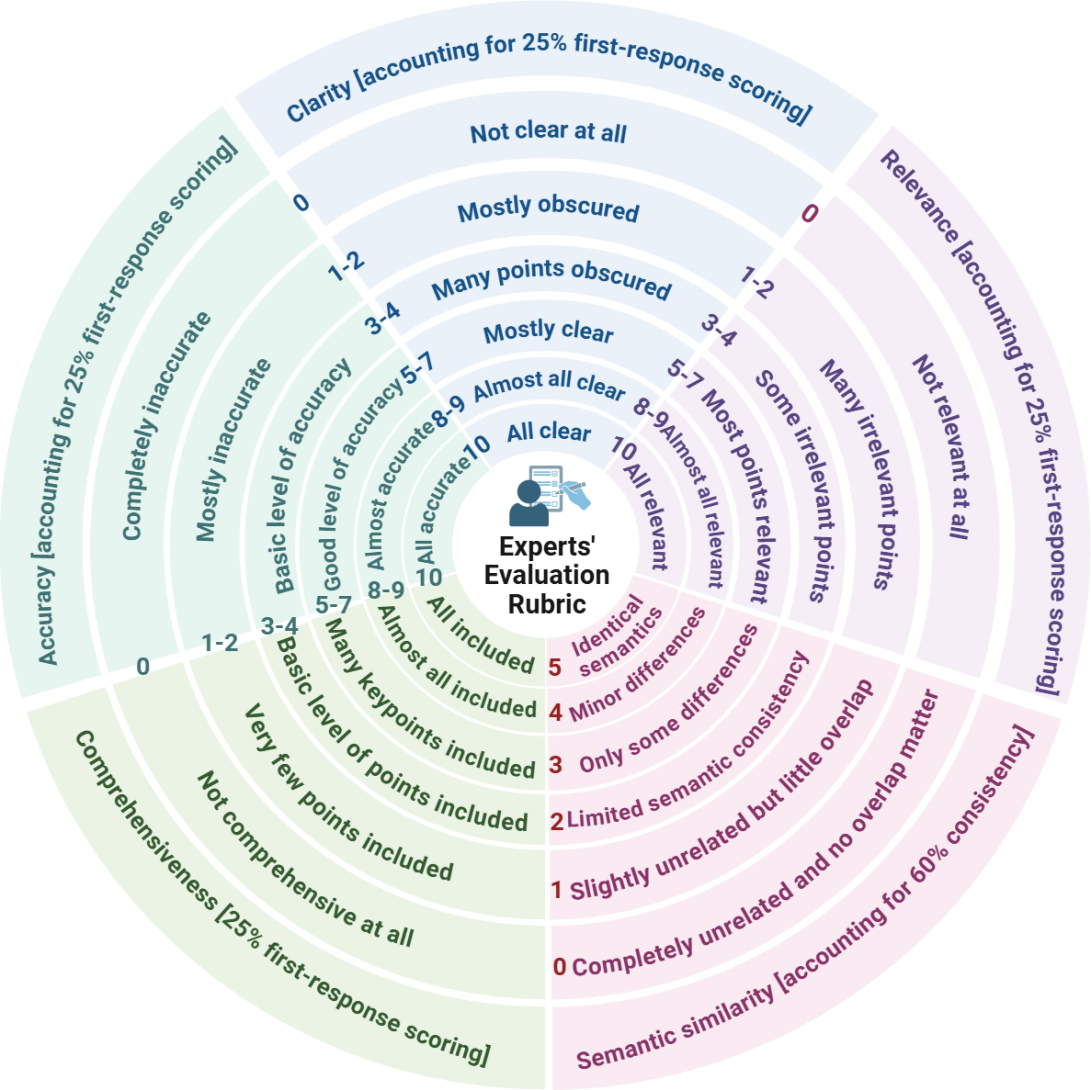
Physician scoring rubric used in the comparative evaluation of model-generated answers for high-altitude public health medical education.

Four LLMs were anonymized and labeled with a code letter (A-D) to ensure blinding during assessment. Sixteen experts participated in the evaluation as 2 independent 8-member panels, one from PUMCH and one from People’s Hospital of Xizang Autonomous Region. All experts were trained and calibrated in advance using the scoring rubric. For each response, the 8 experts within each panel discussed the output and generated one panel-level consensus score using integer ratings from 0 to 10 across 4 predefined dimensions: comprehensiveness, scientific accuracy, relevance, and clarity. Thus, each response yielded 2 consensus scores, one from each panel. The final score for each LLM’s response to a given question was calculated as the average of the 4 dimension scores within each panel; the 2 panel-level consensus scores were then used for interpanel reliability analyses, and their mean was used for subsequent comparative analyses (Figure 3).

### Consistency Analysis

To assess the intramodel stability of the LLMs’ outputs, 3 responses generated for each question were compared using both lexical (word frequency–based) and structural similarity measures. Specifically, for each question, the 3 independently generated responses from the same model were compared in a pairwise manner (response 1 vs response 2, response 1 vs response 3, and response 2 vs response 3), and the resulting similarity scores were averaged across the 3 pairwise comparisons.

For lexical similarity, text content similarity was calculated using term frequency–inverse document frequency vectorization followed by cosine similarity, implemented in Python using third-party libraries including sklearn and numpy. For structural similarity, Python’s built-in difflib.SequenceMatcher was used to quantify structural overlap between texts. In this structural similarity analysis, punctuation marks, spaces, and special characters were ignored so that superficial formatting differences would not disproportionately affect the similarity results. Both lexical similarity and structural similarity ranged from 0 to 1.

However, purely algorithmic similarity metrics have inherent limitations when applied to complex, domain-specific texts such as medical and public health communication. Lexical similarity measures primarily focus on word overlap and frequency, which may overlook paraphrasing, synonym usage, and deeper contextual meaning. Two semantically identical sentences with different wording can thus receive low lexical similarity scores. Similarly, structural similarity metrics, which assess syntactic patterns or sentence structures, may fail to capture differences in the underlying semantic content or clinical implications of responses. These methods cannot reliably detect whether subtle but critical nuances—such as risk factors or treatment recommendations—are consistently conveyed.

Due to these shortcomings, relying solely on algorithmic similarity can misrepresent true consistency and overlook clinically relevant differences. To overcome this, the final consistency evaluation incorporated both quantitative and physician-assessed semantic similarity, which involves expert reviewers evaluating how closely the meaning and clinical relevance of different model-generated answers align, which is fine-tuned with reference to the rubric in SemEval-2017 Task 1 [52]. This expert judgment better captures the practical, real-world equivalence of responses, reflecting nuances that automated metrics miss. Semantic similarity was scored on a 0‐5 scale and subsequently normalized to a 0‐1 scale for integration with the algorithmic metrics. The final composite score was calculated with the following weighting: physician-assessed semantic similarity (60% weight, detailed in Figure 3), structural similarity (20% weight), and lexical similarity (20% weight). This composite score was used to better reflect the meaningful consistency of model responses in clinical and public health communication contexts.

### Development and Evaluation of the New RAG Architecture

Before model output generation, physician scoring, and comparative analysis, the expert panels prospectively agreed to use a clinically weighted composite approach to determine the optimal LLM. Because the first-response score was reported on a 0‐10 scale, whereas the composite consistency metric was presented on a normalized 0‐1 scale, the composite consistency metric was linearly rescaled to a 0‐10 scale before model selection. The final model-selection score was then derived by weighting the first-response score at 60% and the rescaled composite consistency score at 40%. This weighting was selected during study design to reflect the intended real-world use case, in which first-response quality was considered slightly more important than response consistency.

Four widely used authoritative books on high-altitude public health medicine, *Ward, Milledge and West’s High Altitude Medicine and Physiology, High Altitude Medicine: A Case-Based Approach, High Altitude Medicine,* and *High Altitude Medical Protection*, were selected as the external knowledge source for the LLM [53-56]. These authoritative books are essential reading material for individuals entering high-altitude regions and serve as a fundamental reference for medical professionals working in high-altitude medicine in China and around the world. The textbooks were obtained in PDF format from official online sources accessible to the research team. The files were used only in a closed internal research environment to construct and test the retrieval corpus, and no copyrighted content was redistributed or released publicly.

The RAG pipeline was constructed using the LangChain framework, with the text-embedding-ada-002 model used to generate text embeddings. Outputting 1536-dimensional vectors with an 8192-token context window, this model effectively captures the semantic information of medical texts. ChromaDB was used as the vector storage backend. Following chunking, all document segments were transformed into vectors via the embedding model and stored in a local ChromaDB instance to facilitate efficient similarity retrieval. Authoritative textbooks were segmented by chapters and paragraphs using a Recursive Character Text Splitter, with a chunk size of 512 tokens and an overlap of 128 tokens. An initial retrieval of 5 fragments was subsequently reranked, from which the top 3 most relevant segments were selected and fed into the LLM to further mitigate noise. To ensure focused and comparable outputs, we used the identical question set, prompt templates, and hyperparameter settings from the first evaluation phase, configuring only a temperature of 0.1 and a top_p of 0.9, with all other hyperparameters kept at developer defaults.

To reduce expectancy bias in the second-stage evaluation, the assessment of HPHME-Xplus-RAG, which stands for High-Altitude Public Health Medical Education–Xplus (Xiehe in Chinese for Peking Union Medical College Hospital and Xizang for People’s Hospital of Xizang Autonomous Region)–Retrieval-Augmented Generation, was conducted after a washout period of 2 weeks following the initial 4-model evaluation and was coordinated by a study member who was not involved in model development. During this reassessment, the physician panels were informed only that an additional model output set would be evaluated using the same prespecified scoring rubric and consensus procedure as in stage 1. The identity of the system was not disclosed at the time of scoring. Explicit model identifiers and other source-revealing labels were removed from the evaluation materials before assessment. This procedure provided temporal separation and source masking, although it did not constitute an identical fully blinded parallel comparison to the A-D anonymized first-stage assessment.

Following a similar comparative approach, average scores and scores in 4 dimensions of the RAG architecture and selected LLM were compared. To further validate the performance improvement of the RAG architecture over the selected LLM, an additional analysis was conducted to investigate the performance disparity between them across different text-generation scenarios. The 80 questions were partitioned into 2 cohorts based on text generation task types: factual and reasoning questions. Factual questions were defined as those primarily requiring recall or retrieval of explicit information from authoritative sources, whereas reasoning questions were defined as those requiring integration, inference, or application of knowledge to generate the answer (the group label for each question is provided in Multimedia Appendix 1). A subsequent comparative evaluation was performed within each cohort.

### Statistical Analysis

Descriptive statistics, including measures of central tendency (mean and median) and variability (minimum, maximum, SD, SEM, and coefficient of variation), were calculated for each model’s performance. Interpanel reliability was evaluated on the 2 panel-level consensus scores using Spearman ρ, Cronbach α, and the intraclass correlation coefficient (ICC). ICC single and ICC average were calculated using a 2-way mixed-effects model to assess the agreement of a single panel-level consensus score and the reliability of the mean of the 2 panel-level consensus scores, respectively. To test for statistically significant differences between models, the Friedman test followed by the Dunn multiple comparisons test and the paired Wilcoxon signed-rank test were used. In addition to *P* values, we reported effect sizes to quantify the magnitude of differences. For Friedman tests, Kendall W was calculated as a measure of concordance and effect size (Friedman statistic/n(k–1)). For paired Wilcoxon signed-rank tests between HPHME-Xplus-RAG and DeepSeek R1, matched-pairs rank-biserial correlation (r_rb, (R_+_ – R_-_) / (R_+_+R_-_)) was reported. Unless otherwise indicated, nonnormally distributed continuous variables are presented as median (IQR). All analyses were conducted using IBM SPSS (version 29.0), with exact significance levels determined via Monte Carlo simulation. A 2-sided α level of .05 was used throughout.

### Ethical Considerations

This study was reviewed and approved by the Ethics Committee of PUMCH (approval number I-25PJ1246) and the Ethics Committee of the People’s Hospital of the Xizang Autonomous Region (approval number ME-TBHP-25-KJ-124). Although the primary study materials were LLM-generated responses rather than patient data, the study involved 16 medical specialists as structured evaluators, and therefore formal human subjects research ethics review and approval were obtained. No patients, patient records, protected health information, or identifiable public user data were involved. Before participation, all medical specialists were informed of the study purpose, evaluation procedures, voluntary nature of participation, and intended use of the evaluation results, and all agreed to participate. The approved ethics protocol permitted the use of their evaluation scores for this study without additional consent. To protect privacy and confidentiality, individual evaluator identities were not linked to scoring results, and only aggregated panel-level scores and summary statistics are reported. No monetary or nonmonetary compensation was provided. The paper and supplementary materials contain no identifiable images, screenshots, names, account identifiers, or other information that could identify individual participants or users.

## Results

### Overview and Reliability Verification of the Evaluation Between the Two Evaluation Panels

We queried each of the 4 models independently 3 times with the set of 80 questions on medical education on high-altitude health, and their outputs were collated in Multimedia Appendix 2.

In a simulation of real-world conditions, the models’ first responses underwent a blind assessment by 2 independent evaluation panels. Both panels scored the answers of DeepSeek R1 as the best, followed by the answers of Gemini 3.0 Pro, GPT-5.2, and Tencent HY 2.0 (Table 1). Notably, the minimum score of 1.00 assigned to Tencent HY 2.0 by both evaluation panels corresponded to Question 34. On manual review of the raw output, this response was interpreted as a genuine model failure due to informational and clinical insignificance.

**Table 1.** Descriptive statistics for the panel-level consensus scores assigned to the first responses generated by 4 publicly available large language models in a 2-stage cross-sectional comparative evaluation study on high-altitude public health medical education.

First responses scoring	GPT-5.2		Gemini 3.0 Pro		DeepSeek R1		Tencent HY 2.0	
	Evaluation panel 1	Evaluation panel 2	Evaluation panel 1	Evaluation panel 2	Evaluation panel 1	Evaluation panel 2	Evaluation panel 1	Evaluation panel 2
Minimum	4.75	5.25	6.00	6.50	6.75	6.75	1.00	1.00
Median (IQR)	7.00 (0.3125)	7.00 (0.5)	7.25 (0.75)	7.50 (0.75)	7.50 (0.5)	7.75 (0.5)	6.25 (1.0625)	6.50 (0.5)
Maximum	7.75	7.75	8.00	8.00	8.00	8.00	7.25	7.25
Mean (SD)	6.88 (0.42)	6.94 (0.38)	7.33 (0.43)	7.37 (0.41)	7.53 (0.36)	7.63 (0.35)	6.06 (0.90)	6.36 (0.82)
Coefficient of variance (%)	6.14	5.44	5.81	5.53	4.80	4.54	14.83	12.92

To ensure that the 2 evaluation panels adhered to the same criteria, we assessed interpanel reliability based on the 2 panel-level consensus scores for the answers provided by the 4 LLMs (Table 2). Spearman ρ and corresponding *P* values revealed strong and statistically significant correlations between their scores, suggesting that the first responses of the 4 LLMs were evaluated in the same way. Similarly, Cronbach α, ICC single, and ICC average suggested high reliability. All Cronbach α values were >0.7, suggesting strong internal consistency reliability of the 80 questions on medical education on high-altitude health. ICC single and ICC average values were also calculated using a 2-way mixed-effects model to assess the agreement between the two evaluation panels and the reliability of using the average scores provided by the 2 evaluation panels. All ICCs were statistically significant.

**Table 2.** Interpanel reliability for the panel-level consensus scores assigned to the first responses generated by 4 publicly available large language models in a cross-sectional comparative evaluation study on high-altitude public health medical education.

First responses scoring	Spearman ρ	*P* value	Cronbach α	ICC^a^ single	*P* value	ICC average	*P* value
GPT-5.2	0.789	<.001	0.906	0.822	<.001	0.902	<.001
Gemini 3.0 Pro	0.678	<.001	0.794	0.658	<.001	0.794	<.001
DeepSeek R1	0.764	<.001	0.849	0.712	<.001	0.832	<.001
Tencent HY 2.0	0.778	<.001	0.921	0.805	<.001	0.892	<.001

a ICC: intraclass correlation coefficient.

Furthermore, we computed similar descriptive statistics and rigorously assessed interrater reliability for each of the 4 scoring dimensions, including comprehensiveness, scientific accuracy, clarity, and relevance (Tables S3-S10 in Multimedia Appendix 3). Furthermore, the reliability between the two evaluation panels in each dimension was also sufficient.

### Comparison Among the First Responses of 4 LLMs

An average score was computed across the 2 evaluation panels for each question and LLM. The answers of DeepSeek R1 were scored as the best, followed by the answers of Gemini 3.0 Pro, GPT-5.2, and Tencent HY 2.0 (Table 3).

**Table 3.** Descriptive statistics for the mean first-response scores averaged across 2 independent physician-panel consensus ratings for 4 publicly available large language models evaluated on an 80-question benchmark in high-altitude public health medical education.

First responses scoring	GPT-5.2	Gemini 3.0 Pro	DeepSeek R1	Tencent HY 2.0
Minimum	5.00	6.38	7.00	1.00
Median (IQR)	7.00 (0.375)	7.38 (0.53125)	7.63 (0.5)	6.34 (0.875)
Maximum	7.75	8.00	8.00	7.25
Mean (SD)	6.91 (0.38)	7.35 (0.38)	7.58 (0.33)	6.21 (0.83)
Coefficient of variance (%)	5.54	5.17	4.35	13.34

**Figure 4.**
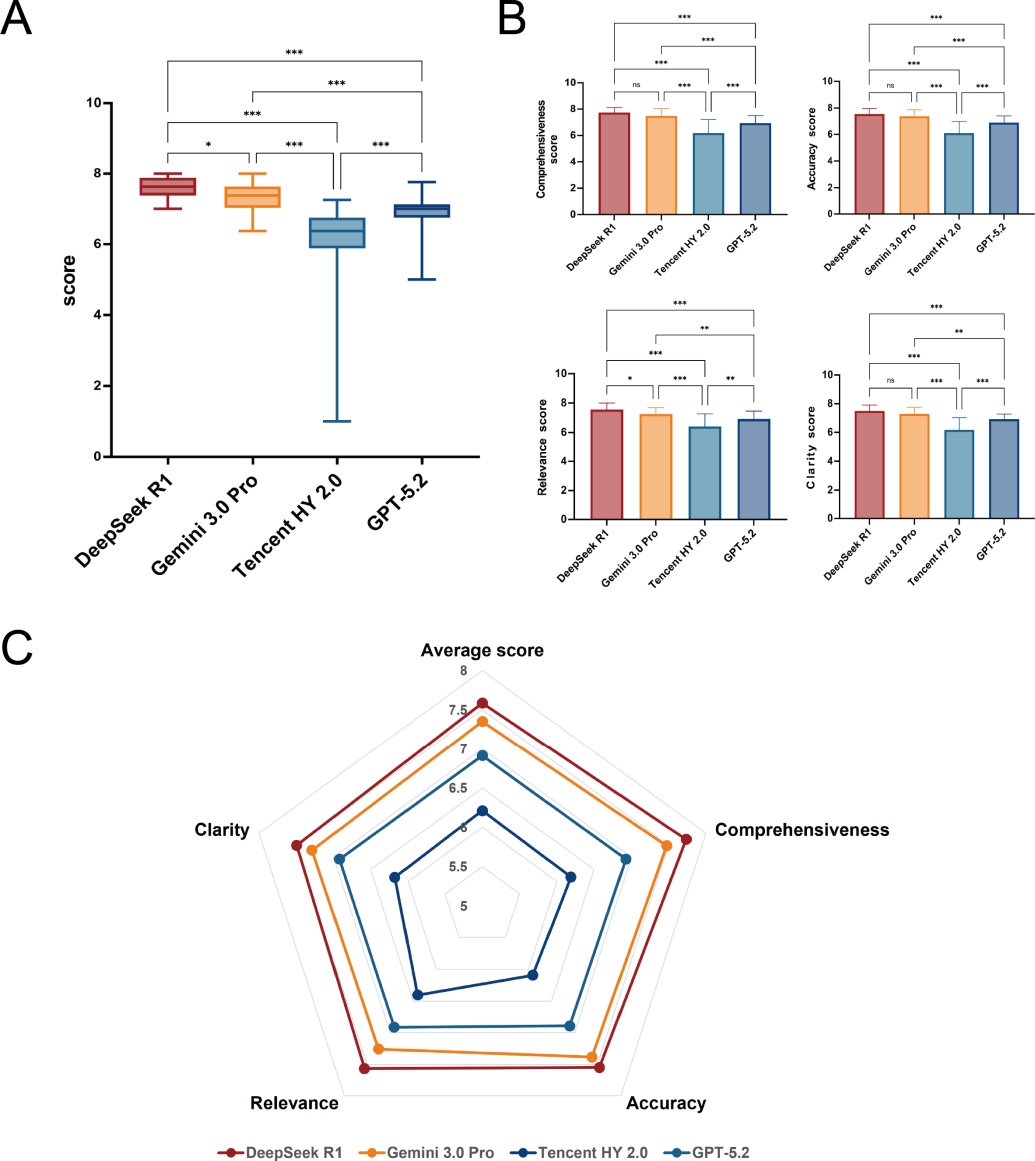
Comparison of first-response performance across 4 publicly available large language models. (A) Overall average scores across the 4 evaluation dimensions. Box plots show the median (center line), IQR (box), and range (whiskers); n=80 questions per model. (B) Mean scores for comprehensiveness, scientific accuracy, relevance, and clarity (bars indicate mean, SD; n=80 per model). (C) Radar plot summarizing overall and dimension-specific performance. *P<.05; **P<.01; ***P<.001; ns, not significant.

Owing to the nonnormal distribution of the rating data, we conducted comparison among the average scores and individual dimension scores of first responses of 4 LLMs by using the Friedman test followed by the Dunn multiple comparisons test (Figure 4). The average scores of the 4 LLMs exhibited strong variation (*P*<.001), with a Kendall W of 0.75, indicating a large overall effect. DeepSeek R1 outperformed the other 3 LLMs (*P*=.01 vs Gemini 3.0 Pro, *P*<.001 vs GPT-5.2 and Tencent HY 2.0), Gemini 3.0 Pro outperformed GPT-5.2st and Tencent HY 2.0 (*P*<.001 vs both), and GPT-5.2 outperformed Tencent HY 2.0 (*P*<.001; Figure 4).

Similarly, the Friedman test revealed significant differences among the average scores of the 4 LLMs in the 4 dimensions (*P*<.001 for all), with Kendall W values of 0.66 for comprehensiveness, 0.99 for scientific accuracy, 0.54 for relevance, and 0.62 for clarity; furthermore, DeepSeek R1 outperformed GPT-5.2 and Tencent HY 2.0 in all the 4 dimensions (*P*<.001 for all), but only outperformed Gemini 3.0 Pro in relevance (*P*=.02) and no significant difference versus Gemini 3.0 Pro in the other 3 dimensions. Gemini 3.0 Pro outperformed GPT-5.2 and Tencent HY 2.0 in all 4 dimensions (*P*<.001 for all except *P*=.006 vs GPT-5.2 in relevance and *P*=.004 vs GPT-5.2 in clarity). GPT-5.2 outperformed Tencent HY 2.0 in all 4 dimensions (*P*<.001 for all except *P*=.002 in relevance; Figure 4). Performance rankings of the average scores held consistent for both the overall score and each of the 4 individual dimensions, with DeepSeek R1 being the best, followed by Gemini 3.0 Pro, GPT-5.2, and Tencent HY 2.0 (Figure 4).

### Comparison of the Consistency of 3 Responses of 4 LLMs

**Figure 5.**
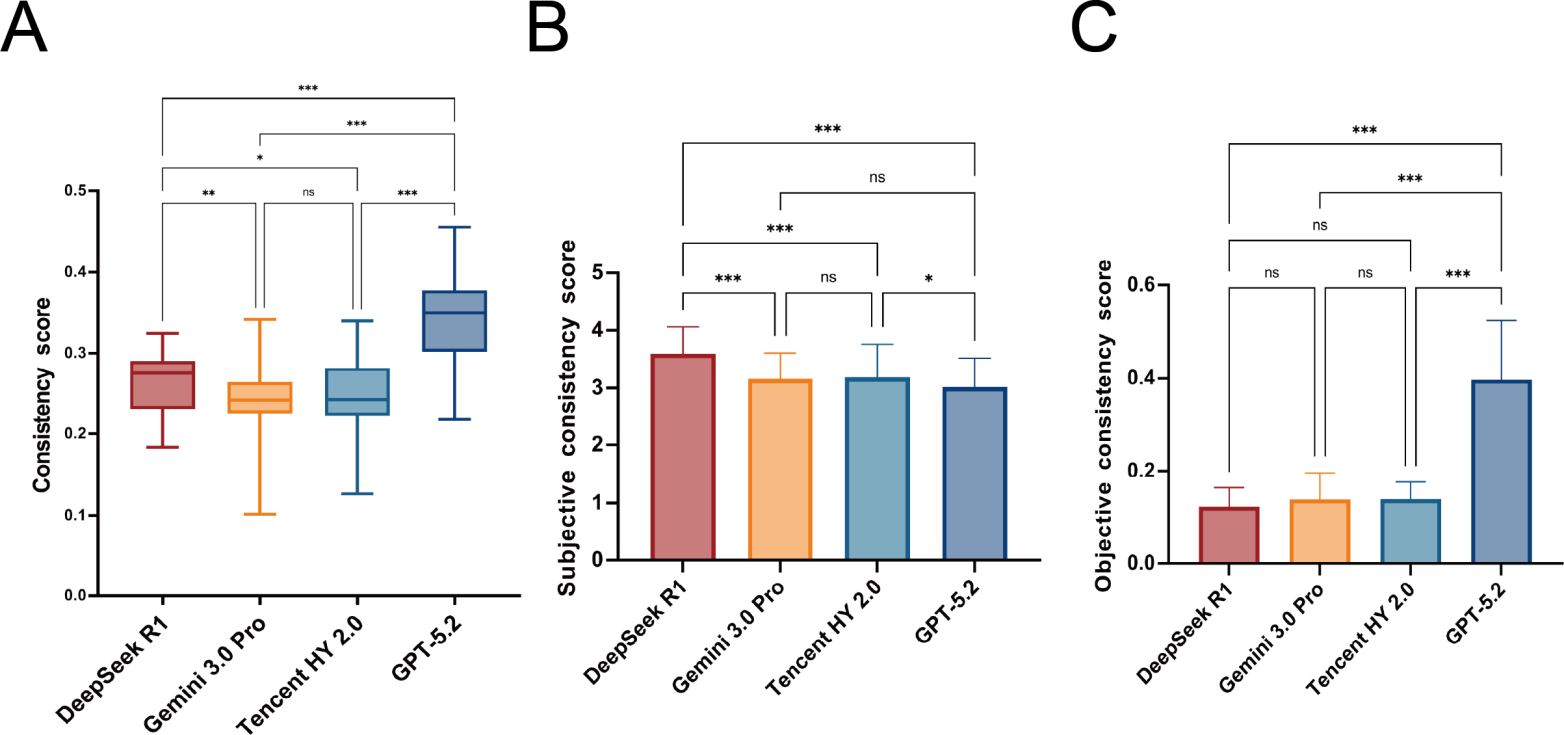
Comparison of response consistency across 4 publicly available large language models. (A) Composite consistency scores across 4 LLMs. Box plots show the median (center line), IQR (box), and range (whiskers); n=80 questions per model. (B) Subjective semantic consistency scores (mean, SD). (C) Objective algorithmic consistency scores (mean, SD). *P<.05; **P<.01; ***P<.001; ns, not significant.

To assess output stability, we measured textual consistency among 3 responses per LLM using a composite consistency index incorporating both subjective semantic and objective algorithmic consistency metrics. Similarly, we adopted the Friedman test followed by the Dunn multiple comparisons test to evaluate disparities in the composite consistency scores. In order to dissect its contributions, the test was also conducted on its 2 subcomponents: the subjective and the objective consistency scores (Figure 5). For the composite consistency, the Friedman test was significant (*P*<.001; Kendall W=0.43, indicating a moderate overall effect). GPT-5.2 achieved the highest consistency, with 0.350 (0.303‐0.376) versus 0.242 (0.227‐0.264) for Gemini 3.0 Pro, 0.275 (0.238‐0.290) for DeepSeek R1, and 0.243 (0.224‐0.281) for Tencent HY 2.0. GPT-5.2 outperformed the other 3 LLMs (*P*<.001 for all). DeepSeek R1 outperformed Tencent HY 2.0 and Gemini 3.0 Pro (*P*=.03 vs Tencent HY 2.0 and *P*=.004 vs Gemini 3.0 Pro). No significant difference was detected between the consistency scores of Tencent HY 2.0 and Gemini 3.0 Pro (Figure 5).

Similarly, Friedman tests revealed strong differences among the subjective and objective consistency scores of the 4 LLMs (*P*<.001 for both, with Kendall W values of 0.24 for subjective consistency scores, 0.57 for objective consistency scores); in comparison of the subjective consistency scores, DeepSeek R1 outperformed the other 3 LLMs (*P*<.001 for all). Tencent HY 2.0 outperformed GPT-5.2 (*P*=.04) and no statistical differences were detected in Gemini 3.0 Pro versus Tencent HY 2.0 and Gemini 3.0 Pro versus GPT-5.2 (Figure 5). As for the comparison of the objective consistency scores, GPT-5.2 outperformed the other 3 LLMs (*P*<.001 for all); however, no statistical differences were detected between the objective consistency scores of the other LLMs (Figure 5).

### Comparison Between the Answers of DeepSeek R1 and HPHME-Xplus-RAG

The top-performing model was selected based on a composite model-selection metric, which combined the first-response score and the composite consistency metric in a 6:4 weighting ratio after the consistency metric had been rescaled from the normalized 0‐1 scale to the 0‐10 scale used for first-response scoring. DeepSeek R1 was finally selected as the best-performing LLM among the 4 LLMs (5.61 vs 5.51 for GPT-5.2, 5.39 for Gemini 3.0 Pro, and 4.71 for Tencent HY 2.0).

With the 4 authoritative books being ingested into a local retrieval corpus for DeepSeek R1, HPHME-Xplus-RAG, a RAG model to support high-altitude public health medical education, was jointly deployed by researchers from PUMCH and People’s Hospital of Xizang Autonomous Region (hereafter referred to as Xplus) [57].

After a 2-week washout period, we additionally queried HPHME-Xplus-RAG with the same 80-question set, and these outputs underwent a source-masked reassessment coordinated by an independent study member; the answers were collated in Multimedia Appendix 2. We computed descriptive statistics and rigorously assessed interrater reliability of the average scores of the answers provided by HPHME-Xplus-RAG and the scores in the 4 dimensions, including comprehensiveness, scientific accuracy, clarity, and relevance (Tables S1-S10 in Multimedia Appendix 3). Also, the reliability between the two evaluation panels was sufficient.

**Figure 6.**
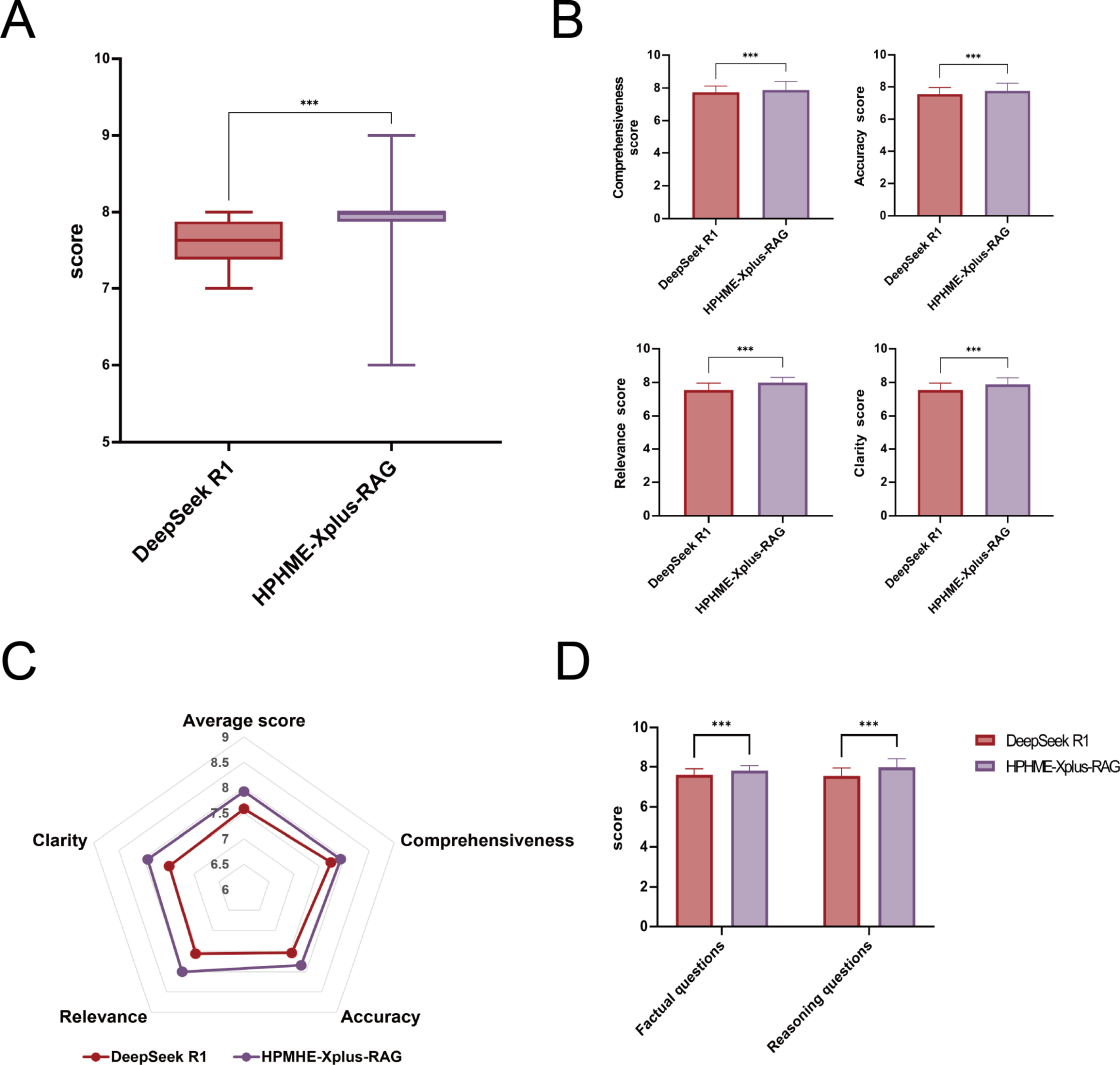
Comparison between the baseline model DeepSeek R1 and HPHME-Xplus-RAG. (A) Overall average first-response scores (box plots show the median, IQR, and range; n=80 questions per model). (B) Mean scores in comprehensiveness, scientific accuracy, relevance, and clarity (bars indicate mean, SD; n=80 per model). (C) Radar plot summarizing overall and dimension-specific performance. (D) Subgroup comparison according to question type, including factual questions (n=51) and reasoning questions (n=29). *P<.05; **P<.01; ***P<.001; HPHME-Xplus-RAG: High-Altitude Public Health Medical Education–Xplus (Xiehe in Chinese for Peking Union Medical College Hospital and Xizang for People’s Hospital of Xizang Autonomous Region)–Retrieval-Augmented Generation; ns, not significant.

We used the paired Wilcoxon signed-rank test to compare the overall ratings between HPHME-Xplus-RAG and DeepSeek R1, which were nonnormally distributed. In addition, the same test was applied to compare ratings within each of the 4 scoring dimensions and the 2 question categories (Figure 6). HPHME-Xplus-RAG achieved higher overall first-response scores (median 8.00 [IQR 7.88‐8.00]) over DeepSeek R1 (median 7.63 [IQR 7.38‐7.88]). The paired Wilcoxon signed-rank test showed that this difference was statistically significant (*P*<.001), with a matched-pairs rank-biserial correlation of r_rb=0.68, indicating a large effect (Figure 6). HPHME-Xplus-RAG outperformed DeepSeek R1 in comprehensiveness (*P*<.001; r_rb=0.60), scientific accuracy (*P*<.001; r_rb=0.62), relevance (*P*<.001; r_rb=0.94), and clarity (*P*<.001; r_rb=0.77), indicating a large effect across all 4 dimensions (Figure 6). The average scores and scores across the 4 dimensions of the answers of HPHME-Xplus-RAG were all higher than those of DeepSeek R1, and the whole improvement was mainly due to the enhancement in clarity and relevance (Figure 6).

To further assess the new model’s performance enhancement in different task scenarios, we divided the question set into 2 groups, factual questions and reasoning questions, and administered paired Wilcoxon signed-rank tests within each group for comparison. Paired Wilcoxon signed-rank tests revealed that HPHME-Xplus-RAG (median 7.88 [IQR 7.75‐8.00] in factual questions and median 8.00 [IQR 7.88‐8.31] in reasoning questions) outperformed DeepSeek R1 (median 7.63 [IQR 7.50‐7.88] in factual questions and median 7.50 [IQR 7.25‐8.00] in reasoning questions in both 2 kinds of questions; *P*<.001 for both question groups; r_rb=0.76 for factual questions and r_rb=0.82 for reasoning questions, both indicating a large effect). And HPHME-Xplus-RAG exhibited a larger performance gain over DeepSeek R1 in reasoning questions compared with factual questions (Figure 6).

## Discussion

### Principal Findings

High-altitude public health medical education is of critical importance in low-resource regions, yet it faces substantial challenges [1-12,14-20]. This study systematically evaluated 4 LLMs (DeepSeek R1, GPT-5.2, Gemini 3.0 Pro, and Tencent HY 2.0) on a set of 80 expert-designed questions covering high-altitude public health medical education. Among the 4 models, DeepSeek R1 achieved the highest weighted composite score, integrating multidimensional first-response performance with composite consistency. Building upon this, we developed HPHME-Xplus-RAG, a RAG-based system integrating 4 authoritative textbooks with DeepSeek R1 as the base model. HPHME-Xplus-RAG demonstrated significant and consistent improvement in overall multidimensional performance compared to baseline DeepSeek R1, confirming that integrating domain-authoritative knowledge via RAG can meaningfully enhance LLM performance in a resource-limited, highly specialized medical domain where specialized model development is neither practical nor cost-effective [30,43,58].

### Comparison to Relevant Literature, Interpretations, and Implications

This study systematically unveils and examines the generative capabilities of LLMs within the domain of high-altitude medicine. Similar to findings reported in other domains, the results of this study strongly support that general LLMs possess the fundamental capability to perform medical education and other tasks; however, their performance remains inconsistent and the overall quality requires further improvement [31-33]. Studies evaluating LLMs in other medical disciplines have also demonstrated the strong performance of the DeepSeek R1 model, while some reports have noted its relatively poorer results in specific specialty tasks [31,34-36]. Differences in model selection, task design, and evaluation results across studies suggest that the capabilities of general LLMs vary across medical specialties and task scenarios. For instance, Tordjman et al [31] demonstrated that DeepSeek R1 achieved an accuracy of 0.92 on the United States Medical Licensing Examination and showed particularly strong diagnostic reasoning performance, though it underperformed relative to ChatGPT-o1 in radiology report summarization. Similarly, a clinical decision-making benchmark involving 125 patient cases found that DeepSeek R1 performed comparably to or better than GPT-4o across diagnosis and treatment recommendation tasks [35].

Consistent with the majority of previous studies, the application of RAG technology led to a significant improvement in model performance within the target domain [59-64]. It is worth noting that this study innovatively used authoritative textbooks as the external knowledge source, providing a new perspective on the selection of authoritative domain knowledge for RAG-based systems and ensuring that authoritative books, which embody knowledge accumulated over generations, can be preserved in a way that keeps pace with the digital era. This approach is particularly applicable to domains lacking formal clinical guidelines, where authoritative textbooks serve as the most reliable and comprehensive knowledge source available.

Notably, RAG significantly improved performance across all 4 evaluated dimensions (Figure 6), indicating that the integration of authoritative textbooks enhanced the overall quality of model responses. However, this improvement was not uniformly distributed across dimensions. The overall gain appeared to be driven primarily by larger improvements in clarity and relevance, although significant benefits were also observed in comprehensiveness and scientific accuracy (Figure 6). This pattern suggests that retrieval from authoritative textbooks may be particularly effective in helping the model generate responses that are better focused, more interpretable, and more closely aligned with the educational intent of the questions, while still strengthening factual grounding. In addition, the larger gain observed for reasoning questions than for factual questions suggests that the retrieved domain knowledge may support not only information recall but also the integration and application of knowledge in more complex response-generation scenarios. Mechanistically, this is consistent with the nature of the external knowledge base used in our RAG pipeline: authoritative textbooks provide systematic, well-structured, and context-rich information that can guide the model toward more coherent and educationally relevant outputs. While the current retrieval pipeline performed well, future work could further explore strategies such as query expansion or multidocument summarization to optimize information extraction for highly complex queries.

Regarding the magnitude of improvement, the observed gain of approximately 0.37 points on the 10-point scale (~4% relative improvement) is modest compared to the larger absolute gains reported in studies using binary accuracy metrics, although the effect is large enough across all dimensions. For example, Ferber et al [60] reported an improvement from 57% to 84% correct responses with RAG applied to oncology guidelines, and Alexandrou et al [64] reported accuracy improvements of approximately 16‐21 percentage points for DeepSeek R1 and ChatGPT-4o on acute coronary syndrome guidelines. However, those studies used binary accuracy outcomes on a different measurement scale, making direct comparison of absolute effect size inappropriate. In addition, the relatively high baseline performance of DeepSeek R1 in our study (median 7.63/10, IQR 0.5) inherently limited the room for further observable improvement. As performance approaches the upper end of the scale, achieving a breakthrough-level absolute increase becomes progressively more difficult, reflecting a typical ceiling effect. Therefore, although the absolute score change was not dramatic, the consistently significant improvements across all evaluated dimensions, together with the large effect sizes, indicate that the benefit of RAG in our setting was both robust and clinically meaningful.

The study goes beyond conventional accuracy assessments by establishing a 4-dimensional evaluation framework encompassing comprehensiveness, scientific accuracy, relevance, and clarity [65]. Although only the models’ first responses were scored to simulate real-world application scenarios, the study innovatively incorporated a consistency evaluation. Rather than relying solely on algorithmic similarity, we proposed a composite consistency metric that integrates quantitative algorithmic indicators with qualitative expert semantic assessments. Furthermore, based on clinical needs, the study adopted a weighted standard that combines first-response scores with composite consistency, providing a more comprehensive and realistic reflection of the models’ reliability and stability in actual clinical settings [65].

In addition, this study applies cutting-edge generative artificial intelligence (AI) technology to a specific, critical, and resource-limited domain—high-altitude medicine—providing a replicable paradigm for AI applications in specialized medical fields. It demonstrates how advanced medical expertise, such as authoritative textbooks, can be rapidly transformed into accessible educational resources through technological innovation. By linking the generation process to authoritative high-altitude medicine literature, the system enhances interpretability and credibility, marking a key step toward the practical integration of AI into clinical and educational workflows.

### Limitations

Several limitations of this study warrant acknowledgment. First, this study focused solely on text generation performance within the context of high-altitude public health medical education; the models’ capabilities across other modalities and task types were not assessed. Second, the entire evaluation was conducted by medical experts, while the intended end users, such as medical instructors and students, frontline health care workers, residents, and travelers, were not directly involved in evaluating system outputs, which may limit the ecological validity of the findings. In addition, the single-system reassessment design cannot fully exclude expectancy effects, even with source masking and temporal separation. Future studies should incorporate real-world user evaluations to complement expert assessments with more rigorous blinded methodology.

Another limitation relates to the benchmark and generalizability of the findings. Although the 80-question benchmark was purposively designed to cover the major domains of high-altitude public health medicine, the selected question set may still not fully capture the breadth and heterogeneity of real-world problems in this field, with an average of approximately 11 questions per domain. Future studies should therefore expand the benchmark to include a broader and more diverse range of clinical, preventive, educational, and public health scenarios, so as to strengthen domain-specific evaluation and real-world generalizability. Moreover, because the benchmark was developed and validated within the specific geographic, cultural, and practice context of the Xizang Autonomous Region, caution is warranted when extrapolating these findings to other low-resource regions, health systems, or medical specialties. Future studies should therefore conduct cross-regional and cross-domain external validation.

Several practical and methodological issues also remain to be addressed. The study does not assess practical deployment considerations such as response latency, cost per query, or system reliability under real-world conditions. Moreover, as LLM application programming interfaces are subject to continuous updates, longitudinal comparisons may be affected by model versioning, complicating reproducibility over time. Finally, the observed score distribution, with maximum values concentrated in the 8‐9 range rather than at the full score of 10, raises the possibility of a ceiling effect. Although the current evaluation framework was sufficient for the aims of this study—being developed through expert discussion, built upon established approaches in the literature, and implemented using a 10-point scale with 0.25-point intervals that provided reasonably fine discrimination—the compression of scores near the upper bound may still limit its sensitivity for distinguishing among very high-performing models. As stronger LLMs continue to emerge, such clustering at the top of the scale may make further absolute improvements harder to detect, even when meaningful qualitative differences exist. Nevertheless, despite using a clinically oriented quantitative framework that combined first-response scoring with composite consistency weighting, the inherent probabilistic nature of LLM outputs cannot be eliminated [43,58]. Future research may therefore benefit from adopting more sensitive rating instruments, such as scales with a wider range, to improve discriminative precision when evaluating increasingly capable systems.

### Conclusions

In summary, this study presents a replicable workflow integrating domain-authoritative knowledge, RAG, and large-model optimization and evaluation for high-altitude public health medical education in the low-resource Xizang Autonomous Region. By integrating authoritative textbooks with an evaluation-optimized general LLM through a RAG framework, the study demonstrated strong performance in a highly specialized educational domain with limited resources. Unlike prior RAG studies that mainly relied on clinical guidelines or focused on diagnosis and treatment tasks, this work used authoritative textbooks for the broader, guideline-scarce field of public health medical education, thereby transforming textbook knowledge into a reliable, specialized, and scalable external knowledge source for LLMs. This approach is particularly suited to highly specialized domains such as high-altitude medicine, where formal clinical guidelines remain limited, and offers a practical way to preserve and deliver authoritative knowledge in the digital era. More broadly, the paradigm of domain-authoritative knowledge+RAG+model optimization and evaluation provides a scalable framework for building trustworthy AI tools in other resource-limited medical and professional settings. With further real-world validation, systems such as HPHME-Xplus-RAG may support clinical and educational practice by providing cost-effective, convenient, and reliable access to medical knowledge.

## Supplementary material

10.2196/92852 Multimedia Appendix 1 The 80-question benchmark with task-type labels and corresponding reference answers used for model evaluation.

10.2196/92852 Multimedia Appendix 2 Outputs of DeepSeek R1, Gemini 3.0 Pro, Tencent HY 2.0, GPT-5.2, and HPHME-Xplus-RAG queried with the 80-question compiled set.

10.2196/92852 Multimedia Appendix 3 Supplementary descriptive statistics and interpanel reliability analyses for overall and dimension-specific scores. The tables include results for the 4 large language models and HPHME-Xplus-RAG.

10.2196/92852 Checklist 1 iCHECK-DH checklist.

## Abbreviations

AI: artificial intelligence
HPHME-Xplus-RAG: High-Altitude Public Health Medical Education–Xplus (Xiehe in Chinese for Peking Union Medical College Hospital and Xizang for People’s Hospital of Xizang Autonomous Region)–Retrieval-Augmented Generation
ICC: intraclass correlation coefficient
LLM: large language model
PUMCH: Peking Union Medical College Hospital
RAG: retrieval-augmented generation
