# Supplementary material for: Authoritative Textbook-Augmented Large Language Models for High-Altitude Public Health Medical Education in the Xizang Autonomous Region: Cross-Sectional Comparative Evaluation Study
Source: J Med Internet Res. 2026 Jun 16;28:e92852. doi: 10.2196/92852 (PMC13271586; doi:10.2196/92852)
Supplement: Multimedia Appendix 3 [file jmir-v28-e92852-s003.docx]

**Multimedia Appendix 3**

**Contents**

**Table S1.** Descriptive statistics for the panel-level consensus scores for the first responses provided by the 4 LLMs and HPHME-Xplus-RAG.

**Table S2.** Spearman’ ρ between the scores, Cronbach α, and ICC for the panel-level consensus scores for the first responses provided by the 4 LLMs and HPHME-Xplus-RAG.

**Table S3.** Descriptive statistics for the panel-level consensus comprehensiveness scores for the first responses provided by the 4 LLMs and HPHME-Xplus-RAG.

**Table S4.** Spearman’ ρ between the scores, Cronbach α, and ICC for the panel-level consensus comprehensiveness scores for the first responses provided by the 4 LLMs and HPHME-Xplus-RAG.

**Table S5.** Descriptive statistics for the panel-level consensus accuracy scores for the first responses provided by the 4 LLMs and HPHME-Xplus-RAG.

**Table S6.** Spearman’ ρ between the scores, Cronbach α, and ICC for the panel-level consensus accuracy scores for the first responses provided by the 4 LLMs and HPHME-Xplus-RAG.

**Table S7.** Descriptive statistics for the panel-level consensus relevance scores for the first responses provided by the 4 LLMs and HPHME-Xplus-RAG.

**Table S8.** Spearman’ ρ between the scores, Cronbach α, and ICC for the panel-level consensus relevance scores for the first responses provided by the 4 LLMs and HPHME-Xplus-RAG.

**Table S9.** Descriptive statistics for the panel-level consensus clarity scores for the first responses provided by the 4 LLMs and HPHME-Xplus-RAG.

**Table S10.** Spearman’ ρ between the scores, Cronbach α, and ICC for the panel-level consensus clarity scores for the first responses provided by the 4 LLMs and HPHME-Xplus-RAG.

Table S1. Descriptive statistics for the panel-level consensus scores for the first responses provided by the 4 LLMs and HPHME-Xplus-RAG.

| First repsonse scoring | GPT-5.2 | | Gemini 3.0 Pro | | DeepSeek R1 | | Tencent HY 2.0 | | HPHME-Xplus-RAG | |
| --- | --- | --- | --- | --- | --- | --- | --- | --- | --- | --- |
|  | Evaluation panel 1 | Evaluation panel 2 | Evaluation panel 1 | Evaluation panel 2 | Evaluation panel 1 | Evaluation panel 2 | Evaluation panel 1 | Evaluation panel 2 | Evaluation panel 1 | Evaluation panel 2 |
| Minimum | 4.75 | 5.25 | 6.00 | 6.50 | 6.75 | 6.75 | 1.00 | 1.00 | 6.25 | 6.00 |
| Median (IQR) | 7.00(0.3125) | 7.00(0.5) | 7.25(0.75) | 7.50(0.75) | 7.50(0.5) | 7.75(0.5) | 6.25(1.0625) | 6.50(0.5) | 8.00(0.25) | 8.00(0.25) |
| Maximum | 7.75 | 7.75 | 8.00 | 8.00 | 8.00 | 8.00 | 7.25 | 7.25 | 9.00 | 8.50 |
| Mean  (SD) | 6.88  (0.42) | 6.94  (0.38) | 7.33  (0.43) | 7.37  (0.41) | 7.53  (0.36) | 7.63  (0.35) | 6.06  (0.90) | 6.36  (0.82) | 7.97  (0.36) | 7.88  (0.31) |
| Coefficient of variance (%) | 6.14 | 5.44 | 5.81 | 5.53 | 4.80 | 4.54 | 14.83 | 12.92 | 4.49 | 3.97 |

Table S2. Spearman ρ between the scores, Cronbach α, and ICC for the panel-level consensus scores for the first responses provided by the 4 LLMs and HPHME-Xplus-RAG.

| First repsonse scoring | Spearman ρ | *P* value | Cronbach α | ICC single | *P* value | ICC average | *P* value |
| --- | --- | --- | --- | --- | --- | --- | --- |
| GPT-5.2 | 0.789 | <.001 | .906 | 0.822 | <.001 | 0.902 | <.001 |
| Gemini 3.0 Pro | 0.678 | <.001 | .794 | 0.658 | <.001 | 0.794 | <.001 |
| DeepSeek R1 | 0.764 | <.001 | .849 | 0.712 | <.001 | 0.832 | <.001 |
| Tencent HY 2.0 | 0.778 | <.001 | .921 | 0.805 | <.001 | 0.892 | <.001 |
| HPHME-Xplus-RAG | 0.513 | <.001 | .824 | 0.683 | <.001 | 0.812 | <.001 |

Table S3. Descriptive statistics for the panel-level consensus comprehensiveness scores for the first responses provided by the 4 LLMs and HPHME-Xplus-RAG.

| Comprehensiveness | GPT-5.2 | | Gemini 3.0 Pro | | DeepSeek R1 | | Tencent HY 2.0 | | HPHME-Xplus-RAG | |
| --- | --- | --- | --- | --- | --- | --- | --- | --- | --- | --- |
|  | Evaluation panel 1 | Evaluation panel 2 | Evaluation panel 1 | Evaluation panel 2 | Evaluation panel 1 | Evaluation panel 2 | Evaluation panel 1 | Evaluation panel 2 | Evaluation panel 1 | Evaluation panel 2 |
| Minimum | 4.00 | 5.00 | 6.00 | 6.00 | 7.00 | 7.00 | 1.00 | 1.00 | 6.00 | 6.00 |
| Median (IQR) | 7.00(0) | 7.00(0) | 8.00(1) | 8.00(1) | 8.00(1) | 8.00(0) | 6.00(1) | 6.00(1) | 8.00(0) | 8.00(0) |
| Maximum | 8.00 | 8.00 | 8.00 | 8.00 | 9.00 | 8.00 | 8.00 | 8.00 | 9.00 | 9.00 |
| Mean  (SD) | 6.88  (0.68) | 6.98  (0.59) | 7.49  (0.60) | 7.46  (0.65) | 7.69  (0.49) | 7.79  (0.41) | 6.05  (1.17) | 6.33  (0.98) | 7.99  (0.52) | 7.88  (0.49) |
| Coefficient of variance (%) | 9.92 | 8.52 | 7.94 | 8.87 | 6.41 | 5.29 | 19.31 | 15.46 | 6.45 | 6.19 |

Table S4. Spearman ρ between the scores, Cronbach α, and ICC for the panel-level consensus comprehensiveness scores for the first responses provided by the 4 LLMs and HPHME-Xplus-RAG.

| Comprehensiveness | Spearman ρ | *P* value | Cronbach α | ICC single | *P* value | ICC average | *P* value |
| --- | --- | --- | --- | --- | --- | --- | --- |
| GPT-5.2 | 0.560 | <.001 | .758 | 0.606 | <.001 | 0.755 | <.001 |
| Gemini 3.0 Pro | 0.571 | <.001 | .708 | 0.551 | <.001 | 0.710 | <.001 |
| DeepSeek R1 | 0.441 | <.001 | .582 | 0.404 | <.001 | 0.575 | <.001 |
| Tencent HY 2.0 | 0.663 | <.001 | .850 | 0.850 | <.001 | 0.835 | <.001 |
| HPHME-Xplus-RAG | 0.529 | <.001 | .748 | 0.586 | <.001 | 0.739 | <.001 |

Table S5. Descriptive statistics for the panel-level consensus accuracy scores for the first responses provided by the 4 LLMs and HPHME-Xplus-RAG.

| Scientific accuracy | GPT-5.2 | | Gemini 3.0 Pro | | DeepSeek R1 | | Tencent HY 2.0 | | HPHME-Xplus-RAG | |
| --- | --- | --- | --- | --- | --- | --- | --- | --- | --- | --- |
|  | Evaluation panel 1 | Evaluation panel 2 | Evaluation panel 1 | Evaluation panel 2 | Evaluation panel 1 | Evaluation panel 2 | Evaluation panel 1 | Evaluation panel 2 | Evaluation panel 1 | Evaluation panel 2 |
| Minimum | 4.00 | 5.00 | 6.00 | 7.00 | 6.00 | 6.00 | 1.00 | 1.00 | 6.00 | 6.00 |
| Median (IQR) | 7.00(0.25) | 7.00(0) | 7.00(1) | 7.50(1) | 7.00(1) | 8.00(1) | 6.00(1) | 7.00(1) | 8.00(0) | 8.00(0) |
| Maximum | 8.00 | 8.00 | 8.00 | 8.00 | 8.00 | 8.00 | 7.00 | 7.00 | 9.00 | 9.00 |
| Mean  (SD) | 6.81  (0.64) | 6.98  (0.48) | 7.26  (0.65) | 7.50  (0.50) | 7.40  (0.52) | 7.69  (0.49) | 5.84  (0.96) | 6.35  (0.92) | 7.89  (0.50) | 7.80  (0.51) |
| Coefficient of variance (%) | 9.40 | 6.83 | 8.96 | 6.71 | 7.00 | 6.41 | 16.45 | 14.42 | 6.38 | 6.58 |

Table S6. Spearman ρ between the scores, Cronbach α, and ICC for the panel-level consensus accuracy scores for the first responses provided by the 4 LLMs and HPHME-Xplus-RAG.

| Scientific accuracy | Spearman ρ | *P* value | Cronbach α | ICC single | *P* value | ICC average | *P* value |
| --- | --- | --- | --- | --- | --- | --- | --- |
| GPT-5.2 | 0.601 | <.001 | .797 | 0.639 | <.001 | 0.780 | <.001 |
| Gemini 3.0 Pro | 0.404 | <.001 | .564 | 0.365 | <.001 | 0.535 | <.001 |
| DeepSeek R1 | 0.366 | <.001 | .515 | 0.301 | .004 | 0.462 | .009 |
| Tencent HY 2.0 | 0.698 | <.001 | .879 | 0.684 | .003 | 0.812 | .005 |
| HPHME-Xplus-RAG | 0.329 | <.001 | .574 | 0.399 | <.001 | 0.571 | <.001 |

Table S7. Descriptive statistics for the panel-level consensus relevance scores for the first responses provided by the 4 LLMs and HPHME-Xplus-RAG.

| Relevance | GPT-5.2 | | Gemini 3.0 Pro | | DeepSeek R1 | | Tencent HY 2.0 | | HPHME-Xplus-RAG | |
| --- | --- | --- | --- | --- | --- | --- | --- | --- | --- | --- |
|  | Evaluation panel 1 | Evaluation panel 2 | Evaluation panel 1 | Evaluation panel 2 | Evaluation panel 1 | Evaluation panel 2 | Evaluation panel 1 | Evaluation panel 2 | Evaluation panel 1 | Evaluation panel 2 |
| Minimum | 5.00 | 5.00 | 6.00 | 6.00 | 6.00 | 6.00 | 1.00 | 1.00 | 7.00 | 6.00 |
| Median (IQR) | 7.00(0) | 7.00(0) | 7.00(1) | 7.00(0.25) | 8.00(1) | 8.00(1) | 6.00(1) | 7.00(1) | 8.00(0) | 8.00(0) |
| Maximum | 8.00 | 8.00 | 8.00 | 8.00 | 8.00 | 8.00 | 8.00 | 8.00 | 9.00 | 9.00 |
| Mean  (SD) | 6.95  (0.61) | 6.88  (0.58) | 7.30  (0.50) | 7.21  (0.50) | 7.61  (0.52) | 7.51  (0.53) | 6.26  (0.99) | 6.55  (0.93) | 8.03  (0.35) | 7.96  (0.35) |
| Coefficient of variance (%) | 8.84 | 8.46 | 7.68 | 6.87 | 6.77 | 7.02 | 15.81 | 14.14 | 4.42 | 4.45 |

Table S8. Spearman ρ between the scores, Cronbach α, and ICC for the panel-level consensus relevance scores for the first responses provided by the 4 LLMs and HPHME-Xplus-RAG.

| Relevance | Spearman ρ | *P* value | Cronbach α | ICC single | *P* value | ICC average | *P* value |
| --- | --- | --- | --- | --- | --- | --- | --- |
| GPT-5.2 | 0.586 | <.001 | .765 | 0.617 | <.001 | 0.763 | <.001 |
| Gemini 3.0 Pro | 0.402 | <.001 | .574 | 0.400 | <.001 | 0.572 | <.001 |
| DeepSeek R1 | 0.412 | <.001 | .585 | 0.409 | <.001 | 0.581 | <.001 |
| Tencent HY 2.0 | 0.448 | <.001 | .780 | 0.615 | <.001 | 0.761 | <.001 |
| HPHME-Xplus-RAG | 0.367 | <.001 | .579 | 0.406 | <.001 | 0.578 | <.001 |

Table S9. Descriptive statistics for the panel-level consensus clarity scores for the first responses provided by the 4 LLMs and HPHME-Xplus-RAG.

| Clarity | GPT-5.2 | | Gemini 3.0 Pro | | DeepSeek R1 | | Tencent HY 2.0 | | HPHME-Xplus-RAG | |
| --- | --- | --- | --- | --- | --- | --- | --- | --- | --- | --- |
|  | Evaluation panel 1 | Evaluation panel 2 | Evaluation panel 1 | Evaluation panel 2 | Evaluation panel 1 | Evaluation panel 2 | Evaluation panel 1 | Evaluation panel 2 | Evaluation panel 1 | Evaluation panel 2 |
| Minimum | 6.00 | 6.00 | 6.00 | 6.00 | 7.00 | 7.00 | 1.00 | 1.00 | 6.00 | 6.00 |
| Median (IQR) | 7.00(0) | 7.00(0) | 7.00(1) | 7.00(1) | 7.00(1) | 8.00(1) | 6.00(1) | 6.00(1) | 8.00(0) | 8.00(0) |
| Maximum | 8.00 | 8.00 | 8.00 | 8.00 | 8.00 | 8.00 | 7.00 | 8.00 | 9.00 | 9.00 |
| Mean  (SD;SE) | 6.90  (0.47,0.05) | 6.94  (0.43,0.05) | 7.28  (0.50,0.06) | 7.30  (0.60,0.07) | 7.44  (0.50,0.06) | 7.55  (0.50,0.06) | 6.13  (0.95,0.11) | 6.23  (0.86,0.10) | 7.96  (0.40,0.05) | 7.89  (0.39,0.04) |
| Coefficient of variance (%) | 6.76 | 6.22 | 6.91 | 8.27 | 6.71 | 6.63 | 15.45 | 13.76 | 5.07 | 4.94 |

Table S10. Spearman ρ between the scores, Cronbach α, and ICC for the panel-level consensus clarity scores for the first responses provided by the 4 LLMs and HPHME-Xplus-RAG.

| Clarity | Spearman ρ | *P* value | Cronbach α | ICC single | *P* value | ICC average | *P* value |
| --- | --- | --- | --- | --- | --- | --- | --- |
| GPT-5.2 | 0.229 | .041 | .360 | 0.221 | 0.024 | 0.362 | .024 |
| Gemini 3.0 Pro | 0.407 | <.001 | .557 | 0.388 | <.001 | 0.560 | <.001 |
| DeepSeek R1 | 0.342 | .002 | .510 | 0.336 | <.001 | 0.503 | <.001 |
| Tencent HY 2.0 | 0.613 | <.001 | .872 | 0.771 | <.001 | 0.870 | <.001 |
| HPHME-Xplus-RAG | 0.508 | <.001 | .762 | 0.608 | <.001 | 0.756 | <.001 |
