# Supplementary material for: Authoritative Textbook-Augmented Large Language Models for High-Altitude Public Health Medical Education in the Xizang Autonomous Region: Cross-Sectional Comparative Evaluation Study
Source: J Med Internet Res. 2026 Jun 16;28:e92852. doi: 10.2196/92852 (PMC13271586; doi:10.2196/92852)
Supplement: Checklist 1 [file jmir-v28-e92852-s004.docx]

Checklist of iCHECK-DH guidelines. iCHECK-DH: Guidelines and Checklist for the Reporting on Digital Health Implementations.

| section |  | Item | Description |
| --- | --- | --- | --- |
| Title | 1 | Title  (M^[[1]](#footnote-1)^) | Authoritative Textbook-Augmented Large Language Models for High-Altitude Public Health Medical Education in the Xizang Autonomous Region: A Comparative Evaluation Study |
| Abstract | 2 | Abstract  (M) | Location in manuscript: Abstract, pages 4-6  What is reported: The abstract summarizes the background, objective, two-stage study design, RAG intervention, evaluation framework, main quantitative results, and conclusions.  If not reported / not applicable: It does not fully report implementation strategy, implementation KPIs, or real-world deployment outputs in the style of a standard digital health implementation report. |
| Introduction | 3 | Context  (M) | Location in manuscript: Introduction, pages 7-8; Methods—Study design, pages 9-10  What is reported: The manuscript describes the low-resource, high-altitude context of the Xizang Autonomous Region, including educational constraints, limited access to standardized resources, and the practical need for high-altitude public health medical education.  If not reported / not applicable: It does not explicitly report alignment with a national digital health strategy or formally classify the implementation stage using WHO Digital Health Atlas terminology. |
|  | 4 | Problem statement  (M) | Location in manuscript: Introduction, pages 7-8  What is reported: The manuscript clearly states the high-altitude public health medical education problem: traditional authoritative textbooks have limited accessibility and interactivity, while general LLMs may hallucinate or perform unreliably in a highly specialized medical domain. |
|  | 5 | Similar Interventions  (M) | Location in manuscript: Introduction, page 8; Discussion—Comparison to Relevant Literature, pages 33-35; Conclusions, page 38  What is reported: The manuscript compares the present work with prior RAG studies and explains that earlier studies mainly relied on clinical guidelines or focused on diagnosis and treatment tasks, whereas this study used authoritative textbooks for public health medical education in a guideline-scarce domain.  If not reported / not applicable: It does not describe the intervention as being directly adapted from one specific prior implementation, so this item is only partially addressed. |
| Methods | 6 | Aims and Objectives  (M) | Location in manuscript: Abstract—Objective/Methods, pages 4-5; Methods—Study design, pages 9-10; Methods—Quantitative Evaluation of First Responses and Consistency Analysis, pages 13-18  What is reported: The manuscript states that the aims were to identify the optimal general-purpose LLM and then evaluate whether integrating authoritative textbooks through RAG improves performance in high-altitude public health medical education. Outcomes were measured using predefined multidimensional first-response scores, a composite consistency metric, and subgroup analyses. |
|  | 7 | Blueprint summary  (M) | Location in manuscript: Methods—Study design, pages 9-10; Methods—Development and evaluation of the new RAG architecture, pages 16-18  What is reported: The manuscript provides a two-stage blueprint: benchmark four general LLMs, select the best-performing base model, integrate it with four authoritative textbooks through a RAG pipeline, and re-evaluate the resulting system using the same benchmark and scoring framework. |
|  | 8 | Technical Design  (M) | Location in manuscript: Methods—Development and evaluation of the new RAG architecture, pages 16-18; Results—Comparison between the answers of DeepSeek R1 and HPHME-Xplus-RAG, page 28; References, page 48  What is reported: The manuscript describes the technical architecture, including LangChain, text-embedding-ada-002, local ChromaDB storage, chunking, reranking, top-3 retrieval, and integration with DeepSeek R1. It also reports that four authoritative textbooks were used as the external knowledge base and provides the system name HPHME-Xplus-RAG.  If not reported / not applicable: The manuscript does not fully report software licensing, code documentation, intellectual property, or health enterprise architecture integration, so this item is only partially covered. |
|  | 9 | Target  (M) | Location in manuscript: Introduction, pages 7-8; Methods—Data collection, pages 10-12; Discussion—Limitations, page 36; Conclusions, pages 38-39  What is reported: The manuscript identifies the target problem and target setting as high-altitude public health medical education in a low-resource region, with potential end users including medical instructors and students, frontline healthcare workers, hospitals, and public health services.  If not reported / not applicable: Detailed site-level implementation characteristics, staffing, resource specifications, or formal eligibility criteria for end users are not provided. |
|  | 10 | Data  (M) | Location in manuscript: Methods—Development and evaluation of the new RAG architecture, pages 16-18; Ethical Considerations, page 20; Data availability, page 40  What is reported: The manuscript reports that the external knowledge base consisted of four authoritative textbooks obtained from official online sources and used in a closed internal research environment; vectorized text was stored locally in ChromaDB. It also clarifies which study materials are publicly shareable and which copyrighted textbook contents cannot be redistributed.  If not reported / not applicable: The manuscript does not provide a full data governance framework covering data life cycle, cybersecurity, ownership, legal compliance, or patient consent. Patient consent was not applicable because no patient-level human subject data were used. |
|  | 11 | Interoperability  (M) | Location in manuscript: Not specifically reported  What is reported: No dedicated interoperability section is provided.  If not reported / not applicable: The study evaluated a closed research prototype and did not report integration with electronic health records, hospital information systems, or interoperability standards such as HL7 FHIR, SNOMED, or LOINC. |
|  | 12 | Participating entities  (M) | Location in manuscript: Author affiliations, pages 1-3; Methods—Data collection, pages 10-12; Results—Comparison between the answers of DeepSeek R1 and HPHME-Xplus-RAG, page 28; Funding, page 39; Authors’ contributions, page 40  What is reported: The manuscript identifies the main participating institutions, including Peking Union Medical College Hospital and People’s Hospital of Xizang Autonomous Region, and describes their collaboration in question development, evaluation, and system deployment. Funding sources and author contributions are also provided.  If not reported / not applicable: It does not fully describe organizational missions, leadership, government involvement by implementation stage, or final intellectual property ownership. |
|  | 13 | Budget Planning  (M) | Location in manuscript: Not specifically reported; related limitation noted in Discussion—Limitations, page 37  What is reported: No implementation budget, cost breakdown, training cost, maintenance cost, or total cost of ownership is reported.  If not reported / not applicable: This was a comparative evaluation study and prototype development rather than a formal implementation program with budget reporting. |
|  | 14 | Sustainability  (M) | Location in manuscript: Discussion—Comparison to Relevant Literature, pages 33-35; Conclusions, pages 38-39; Discussion—Limitations, page 37  What is reported: The manuscript discusses the scalability, transferability, and practical potential of the workflow in resource-limited settings and notes that such systems may provide cost-effective and trustworthy access to medical knowledge after further real-world validation.  If not reported / not applicable: It does not report a business model, long-term maintenance plan, institutionalization strategy, or formal sustainability assessment. |
| RESULTS | 15 | Coverage  (M) | Location in manuscript: Introduction, pages 7-8; Results—Comparison between the answers of DeepSeek R1 and HPHME-Xplus-RAG, page 28; Conclusions, pages 38-39  What is reported: The manuscript clearly situates the work in the Xizang Autonomous Region, a subnational high-altitude and low-resource setting, and states that the system was jointly developed/deployed by collaborating institutions serving that setting.  If not reported / not applicable: It does not quantify implementation coverage such as the number of sites, number of users, or percentage of the eligible population reached. |
|  | 16 | Outcomes  (M) | Location in manuscript: Abstract—Results, page 5; Results, pages 21-30  What is reported: The manuscript reports the predefined quantitative outcomes, including first-response performance across four dimensions, inter-panel reliability, composite consistency, weighted model-selection results, and the comparative improvement of HPHME-Xplus-RAG over baseline DeepSeek R1, including subgroup analyses. |
|  |  | Lessons learned  (M) | Location in manuscript: Discussion—Comparison to Relevant Literature, pages 33-35; Discussion—Limitations, pages 36-37; Conclusions, pages 38-39  What is reported: The manuscript identifies several key lessons: authoritative textbooks can serve as an effective knowledge source for RAG in guideline-scarce domains; performance gains were especially notable in clarity, relevance, and reasoning tasks; and future work should improve external validation, benchmark breadth, and rating sensitivity.  If not reported / not applicable: A standalone “lessons learned” subsection is not provided, and implementation challenges, budget adherence, and operational lessons are not described in the detail expected for a full implementation report. |
|  | 17 |  |  |
|  | 18 | Unintended consequences  (NM^[[2]](#footnote-2)^) | Location in manuscript: Not specifically reported  What is reported: No unintended consequences, harms, or side effects are reported.  If not reported / not applicable: The study was a benchmark-based comparative evaluation rather than a real-world deployment study, so unintended consequences were not systematically assessed. |
| Discussion | 19 | Conclusion  (M) | Location in manuscript: Conclusions, pages 38-39  What is reported: The conclusion summarizes the main findings, emphasizes the value of integrating authoritative textbooks with an evaluation-optimized LLM through RAG, and highlights the broader implications of this workflow for other resource-limited medical and professional domains |
| General | 20 | General  (NM) | Location in manuscript: Ethical Considerations, page 20; Funding, page 39; Conflicts of Interest, page 40; Data availability, page 40  What is reported: The manuscript includes statements on ethics, funding, conflicts of interest, and data availability. It states that ethical approval was not required because the study did not involve human or animal subjects, and it clarifies what materials are publicly available and what copyrighted content cannot be shared.  If not reported / not applicable: Trial registration and protocol registration are not reported because the study was not a clinical trial or prospectively registered implementation study. |

1. M: Mandatory item [↑](#footnote-ref-1)
2. NM : Non-mandatory item [↑](#footnote-ref-2)
